# Rates of molecular evolution and diversification in plants: chloroplast substitution rates correlate with species-richness in the Proteaceae

**DOI:** 10.1186/1471-2148-13-65

**Published:** 2013-03-13

**Authors:** David Duchene, Lindell Bromham

**Affiliations:** 1 Centre for Macroevolution and Macroecology, Evolution, Ecology & Genetics, Research School of Biology, Australian National University, Canberra, ACT, 0200, Australia

**Keywords:** Phylogenetic, Substitution, Comparative analysis, Synonymous, Nonsynonymous, dN/dS, Reproductive isolation, Incompatibility

## Abstract

**Background:**

Many factors have been identified as correlates of the rate of molecular evolution, such as body size and generation length. Analysis of many molecular phylogenies has also revealed correlations between substitution rates and clade size, suggesting a link between rates of molecular evolution and the process of diversification. However, it is not known whether this relationship applies to all lineages and all sequences. Here, in order to investigate how widespread this phenomenon is, we investigate patterns of substitution in chloroplast genomes of the diverse angiosperm family Proteaceae. We used DNA sequences from six chloroplast genes (6278bp alignment with 62 taxa) to test for a correlation between diversification and the rate of substitutions.

**Results:**

Using phylogenetically-independent sister pairs, we show that species-rich lineages of Proteaceae tend to have significantly higher chloroplast substitution rates, for both synonymous and non-synonymous substitutions.

**Conclusions:**

We show that the rate of molecular evolution in chloroplast genomes is correlated with net diversification rates in this large plant family. We discuss the possible causes of this relationship, including molecular evolution driving diversification, speciation increasing the rate of substitutions, or a third factor causing an indirect link between molecular and diversification rates. The link between the synonymous substitution rate and clade size is consistent with a role for the mutation rate of chloroplasts driving the speed of reproductive isolation. We find no significant differences in the ratio of non-synonymous to synonymous substitutions between lineages differing in net diversification rate, therefore we detect no signal of population size changes or alteration in selection pressures that might be causing this relationship.

## Background

Present biodiversity has come about through processes of diversification and extinction of species, and the mechanisms that drive these processes are a central focus in evolutionary biology (e.g. [1-4]). One intriguing relationship that has been revealed through studies of branch lengths on molecular phylogenies is a link between the rate of molecular evolution and the net diversification rate. A correlation between evolutionary rates and species diversity has been found in several groups including flowering plants [5,6], reptiles [7], birds [7,8], and other metazoan phyla, orders, and classes [9].

**Table 1:** Genera of sister clades, and their corresponding species richness and branch length estimates

**Pair**	**Sister clades**	**Taxa**	**Species richness**	**Genes available**	**Total branch lengths**	**dN branch lengths**	**dS branch lengths**	**dN/dS**
1	Persoonia	*Persoonia spp.*	100	2	0.00303	0.00331	0.00299	1.10704
	Garnieria + Acidonia	*Garnieria spathulaefolia*	2		0.00097	0.00105	0.00170	0.61562
2	Symphionema	*Symphionema montanum*	2	4	0.01329	0.00990	0.01713	0.57818
	Agastachys	*Agastachys odorata*	1		0.00828	0.00403	0.02123	0.18971
3	Cenarrhenes + Dilobeia	*Cenarrhenes nitida*	3	1	0.01703	0.00379	0.02849	0.13315
	Beaupreopsis	*Beaupreopsis paniculata*	1		0.00936	0.00315	0.01317	0.23892
4	Conospermum + Synaphea	*Conospermum spp.*	104	4	0.03480	0.01312	0.06744	0.19454
	Stirlingia	*Stirlingia latifolia*	7		0.02865	0.01164	0.05841	0.19922
5	Protea	*Protea cynaroides*	112	5	0.00872	0.00588	0.01364	0.43081
	Faurea	*Faurea spp.*	15		0.00660	0.00589	0.01052	0.55959
6	Petrophile	*Petrophile spp.*	53	3	0.01424	0.00723	0.02834	0.25512
	Aulax	*Aulax spp.*	3		0.01537	0.01279	0.02951	0.43337
7	Paranomus	*Paranomus spp.*	19	2	0.00130	0.00187	1.00E-09	1.87E + 06
	Vexatorella	*Vexatorella alpina*	4		0.00130	0.00047	0.00445	0.10504
8	Leucospermum	*Leucospermum spp.*	48	2	0.00129	0.00095	0.00292	0.32577
	Orothamnus + Diastella + Mimetes	*Mimetes spp.*	21		0.00183	0.00187	0.00296	0.63319
9	Alloxylon + Oreocallis	*Alloxylon spp.*	6	4	0.00641	0.00418	0.00931	0.44871
	Embothrium	*Embothrium coccineum*	1		0.00577	0.00323	0.00625	0.51730
10	Stenocarpus + Strangea	*Stenocarpus salignus*	26	6	0.01094	0.00602	0.01962	0.30693
	Lomatia	*Lomatia spp.*	12		0.00625	0.00395	0.01304	0.30245
11	Grevillea + Finschia + Hakea	*Grevillea spp.*	515	6	0.01847	0.01027	0.03031	0.33881
	Buckinghamia	*Buckinghamia spp.*	2		0.00306	0.00118	0.00916	0.12925
12	Virotia	*Virotia leptophylla*	6	1	0.00091	0.00097	1.00E-09	9.71E + 05
	Athertonia	*Athertonia diversifolia*	1		0.00754	0.00535	0.01243	0.43028
13	Panopsis + Brabejum	*Panopsis spp.*	27	4	0.00966	0.00577	0.01995	0.28920
	Macadamia	*Macadamia spp.*	9		0.00752	0.00623	0.01371	0.45473
14	Hicksbeachia	*Hicksbeachia pinnatifolia*	2	1	0.00229	0.00261	0.00095	2.75046
	Gevuina	*Gevuina avellana*	1		0.00220	0.00098	0.00309	0.31894
15	Euplassa	*Euplassas occidentalis*	20	3	0.00318	0.00281	0.00448	0.62802
	Sleumerodendron + Kermadecia + Turrillia	*Sleumerodendron austrocaledonicum + Kermadecia pronyensis*	8		0.00262	0.00199	0.00619	0.32064
16	Banksia + Dryandra	*Banksia spp.*	169	6	0.01403	0.00893	0.02906	0.30745
	Austromuellera + Musgravea	*Austromuellera trinervia*	4		0.01107	0.00525	0.02498	0.21017
17	Roupala + Neorites	*Roupala montana + Roupala monosperma + Neorites kevediana*	34	6	0.00620	0.00286	0.01367	0.20891
	Orites	*Orites spp.*	8		0.00420	0.00229	0.00483	0.47439
18	Darlingia	*Darlingia darlingiana*	2	2	0.00219	0.00236	0.00295	0.79752
	Floydia	*Floydia praealta*	1		0.00297	0.00132	0.00571	0.23042
19	Lambertia	*Lambertia spp.*	10	4	0.02536	0.01116	0.05726	0.19491
	Xylomelum	*Xylomelum spp.*	6		0.02419	0.01036	0.04448	0.23296
20	Helicia	*Helicia spp.*	97	3	0.00149	0.00094	0.00304	3.10E-01
	Hollandaea	*Hollandaea riparia*	4		0.00039	0.00000	0	0

Detailed legend: Sister comparisons included in this analysis. For each pair, we list the two clades compared (a “+” indicates where more than one genus were combined as one sister lineage of a comparison). The taxon from which the sequence was taken is listed, with “spp” indicating that several congeneric sequences were combined (see Table S1 for details). The species richness for each sister lineage is taken from Sauquet et al. (2009), and the number of available gene sequences for each comparison, out of the six chloroplast genes analysed in this study (See Additional file 1: Table S1). The estimated substitution rates for each sister lineage are given for Total branch lengths (all substitutions) as well as synonymous (dS) and non-synonymous (dN) substitutions, and estimates of ω (dN/dS).

However, not all the datasets analysed have provided evidence for a link between diversification rates and rates of molecular evolution. One study of a large number of phylogenies found a relationship between root-to-tip branch lengths and net diversification in around half of the phylogenies tested, but it is not clear whether this was due to low power or lack of a common trend [10]. Another study examined genetic data within the mammals and found no evidence of an association between molecular rates and net diversification [11]. Accordingly, the universality and causes of the link remain uncertain.

There are three possible causes for the association between net diversification and the rate of molecular evolution [5,6]. One is that the process of diversification drives changes in the rate of molecular evolution. Speciation might influence the rate of molecular evolution through positive selection on particular genes associated with adaptation to novel niches [12]. Speciation could also cause genome-wide increases in substitution rate if speciation is typically associated with population subdivision [13,14]. This is because a reduction in effective population size (*N*_*e*_) can cause a higher rate of fixation of nearly neutral mutations (e.g. [15]), leading to a faster substitution rate [14].

Conversely, a higher rate of molecular evolution may increase the diversification rate. A faster mutation rate may hasten differentiation between populations and promote reproductive incompatibility [16]. For example, it has been suggested that higher standing genetic diversity in populations at low latitude may contribute to faster diversification in the tropics [17]. Increased standing genetic variation may produce more raw material for adaptation [18] or reduce the likelihood of extinction [19]. However, a recent study of orchids found no evidence for a link between population genetic variability and net diversification rate [20]. A higher rate of molecular evolution may increase the rate of diversification by accelerating the formation of hybrid incompatibility, occurring through the accumulation of genetic incompatibilities between the genomes of the diverging populations [16].

Alternatively, there might be a third factor that influences both the rate of molecular evolution and diversification rate, creating an indirect link between diversification and molecular evolution. For example, environmental energy (temperature and UV light) has been associated with both the diversification rate and the rate of molecular evolution in angiosperms [21]. Other potential third factors are life history features, such as size or generation length, which are linked with the rate of molecular evolution and diversification rates of angiosperms and several metazoan taxa [9,22-24]. It has also been suggested that both morphological and molecular rates of change may be connected to diversification rate [24]. Whether the correlation between rate of molecular evolution and net diversification has a causal or indirect effect needs more investigation.

One way of disentangling the potential causes of the observed relationship between diversification rate and rate of molecular evolution is to partition substitutions in protein-coding genes into synonymous and non-synonymous substitutions. Synonymous mutations do not change the amino acid sequence of a protein and hence are expected to behave as neutral. If so, then the synonymous substitution rate (dS) should reflect only the mutation rate [25]. Nonsynonymous mutations are expected to have a range of fitness effects, including neutral, positive and negative, so may be subject to both drift and selection. An increase in the nonsynonymous substitution rate (dN) relative to the synonymous rate (dS) can occur through positive selection promoting the fixation of nonsynonymous mutations, or through a reduction in population size increasing the rate of fixation of nearly neutral mutations by drift.

The link between rate of molecular evolution and diversification rate has been attributed to the action of selection during speciation, or to a reduction in average population size in lineages undergoing diversification [14], both of which would be expected to increase the relative rate of nonsynonymous substitutions. However, studies in angiosperms [6], reptiles [7], and birds [7,8] have found a correlation between synonymous substitutions and net diversification, leading to the suggestion that the link between molecular rates and net diversification may be driven by the mutation rate.

Here, we focus on the rate of molecular evolution in chloroplast genes. Genetic changes in chloroplast genomes have been implicated in the process of speciation in plants. Coevolution between organelle and nuclear genomes has been recognized as an important factor in plant diversification [26]. Plastome-genome incompatibility can cause hybrid sterility or inviability, by disrupting sexual reproduction, leaf morphologies, and machineries for photosynthesis or respiration [27-29]. Some of the genetic events in chloroplasts that produce these aberrations are gene duplications, loss of gene complexes and genome rearrangements [26,30,31]. The resulting incompatibilities are probably generalized phenomena in plants, and the evolutionary consequence is that they can enhance post-zygotic barriers during speciation [26,29,32,33]. It seems possible, then, that variation in rates of molecular evolution of chloroplasts could also influence the speed of genetic isolation, and hence the diversification rate of plant lineages.

Using a phylogenetic comparative analysis of sister pairs [34], we investigated the relationship between rates of molecular evolution and net diversification in chloroplast genes of the plant family Proteaceae. This highly diverse family is mostly restricted to the Southern Hemisphere. It contains 79 recognized genera and around 1600 species, and some of its most diverse groups are the Australian genus *Banksia* and the African genus *Protea*. The high diversity of Proteaceae makes it a particularly attractive case study for diversification (e.g. [35-37]). In addition, the family has stark contrasts in species-richness between genera even within its biodiversity hotspots [38]. Of particular interest to this study are the numerous cases of monophyletic sister clades with remarkable differences in number of species. For example, the genus *Protea* has 112 species, while its sister genus *Faurea* has 15, and the *Banksia* lineage (including the dryandras) has 169 species while its sister lineage of the genera *Austromuellera* and *Musgravea* contains only 4.

We focus on the rates of evolution of six chloroplast genes available for a genus level phylogeny of the family Proteaceae [38]. We use three protein-coding genes to estimate and contrast rates of synonymous (dS) and non-synonymous (dN) substitutions. Comparing dN, dS, and ω (dN/dS) to species-richness of clades allows us to separate the effect of mutation rate on net diversification from the effect of selection and effective population size. In this way, we aim to provide insight into the factors underlying the correlation between rates of molecular evolution and net diversification.

## Results

A model where every branch in the phylogeny had an independent rate of substitutions had a significantly higher likelihood than the constant rates model in all the rates estimations (all substitutions, dN, dS), and ω (P value < 0.01 for all tests; see Methods section), indicating that the rate of molecular evolution of the chloroplast genes analysed varies significantly between lineages of the family Proteaceae.

Species-rich lineages had significantly longer branch lengths in the phylogeny estimated from the full 6-gene dataset (one-tailed Wilcoxon Signed-Rank test, W = 175, P = 0.0036). This is evidence of a positive association between net diversification and the rate of molecular evolution of chloroplasts in the family Proteaceae. We also found significant differences in estimates of synonymous (dS: W = 152, P = 0.041), and non-synonymous rates (dN: W = 165, P = 0.012). However, we did not find a significant differences in estimates of ω between species-rich and species-poor sister lineages (W = 100, P = 0.14).

## Discussion

We found a significant positive association between the rate of molecular evolution in chloroplast genes and species-richness in the plant family Proteaceae. There were significant associations between both synonymous and non-synonymous rates of substitutions and net diversification, but not between ω (dN/dS) and diversification. The pattern of correlations in this study are consistent with other studies of angiosperms [6,39], reptiles, and birds [7,8,10]. Importantly, our results give some insight into the cause of this relationship. The variation in both synonymous and non-synonymous substitution rates between lineages may reflect a role for the rate of production of mutations in the chloroplast genome in the process of diversification in the Proteaceae. Because we fail to detect an increase in ω in species-rich clades, our analysis provides no tangible evidence for a role of selection or population size change in driving the relationship between substitution rates and diversification rates in this group.

### Synonymous substitutions and net diversification

Synonymous substitution rates are typically interpreted to reflect the rate of production of mutations. Mutation rates are known to vary between lineages for a range of reasons. For example, species with shorter generation times tend to have faster mutation rates [23], presumably due to the accumulation of DNA replication errors [40]. Mutation rates can also vary across the genome, which may be at least in part due to differences in base composition or gene length [24,41].

Since synonymous substitutions are commonly assumed to be functionally neutral, they are often used to provide a window into variation of mutation rates. However, bias in codon use can influence the synonymous rate if, for example, there is selection for efficiency in the process of translation [42]. This type of bias has been found in angiosperm mitochondrial genes although with selection that is so weak that it is considered not to affect estimations of mutation rates [43]. The chloroplast genome of angiosperms also has minimal codon bias and weak selection for translation efficiency [44]. Therefore, in this study, we consider that the relationship between synonymous substitution rate and net diversification is telling us something about the link between mutation and diversification, whether it reflects differences in the absolute mutation rate per unit time or in the differences in the distribution of fitness effects of synonymous mutations between lineages.

One explanation for the link between synonymous substitutions and net diversification is that higher mutation rates could cause faster genetic divergence between lineages. In this case, genes of chloroplast origin may be important because they can drive reproductive isolation in plants by interacting with nuclear alleles [25]. Reproductive barriers can occur due to the failure of interactions between nuclear and cytoplasmic gene complexes, for example cytoplasmic male sterility [45]. An increased mutation rate may generate more molecular changes that cause these phenomena, known as Bateson-Dobzhansky-Muller (BDM) incompatibilities, and so might accelerate post-zygotic isolation [16,46,47].

Some studies have found that lineage-specific variation in rates of molecular evolution are consistent across the nuclear, mitochondrial, and chloroplast genomes [48], so it may be that the increase in substitution rates that we detected also apply to the nuclear genomes of species-rich lineages in the Proteaceae. In this case, higher rates of substitution in the nuclear genome may be contributing to the formation of incompatibilities between diverging populations, either by generating BDM incompatibilities between the nuclear genomes or through interactions between the nuclear and organelle genomes.

Therefore, the association between the synonymous rate of chloroplast genes and diversification rate reported here may reflect the acceleration in the formation of post-zygotic reproductive isolation. This is also consistent with our finding of an association between non-synonymous rates and net diversification because an increase in the mutation rate should also result in more effectively neutral non-synonymous substitutions going to fixation.

### Indirect links between diversification and the rate of molecular evolution

An indirect relationship between the rate of molecular evolution and diversification could arise if some factor influenced both. For example, it has been suggested that tropical lineages have a higher rate of molecular evolution than their temperate counterparts [49]. This correlation might reflect a direct effect of temperature or UV light on mutagenesis [50], or an indirect effect if higher environmental energy leads to further growth rates and more rapid generation turnover, which could influence the mutation rate through accumulation of replication errors [23]. If higher growth rates also lead to faster diversification [51], then this could create an indirect link between the mutation rate and diversification. This may also explain the patterns in a study on angiosperms that investigated the correlations between species-richness, the rate of molecular evolution, and three energy variables (temperature, UV light, and evapotransportation), but which found no support for the mutation rate as the direct mediator of species-richness [21]. However, it is interesting to note that the Proteaceae do not appear to have higher rates of diversification in the tropics. Instead, much of their radiation has occurred in Mediterranean climate hotspots [38].

Life history variation provides another possible indirect link between rates of molecular evolution and diversification. Several studies have suggested that annual plants have a faster rate of molecular evolution than perennials, a pattern generally attributed to the generation time effect (see [24,40]). The potential for interactions between mechanisms that influence species-richness and the rate of molecular evolution has a broad scope and remains to be studied in detail.

### Net diversification and ω

It has been suggested that processes associated with speciation drive the link between rates of substitution and net diversification [10,13,14], including diversifying selection and changes in effective population size. A reduction in effective population size (*N*_*e*_) may be caused by a speciation event that changes the population structure, such as vicariant or peripatric speciation [52]. This could lead to new adaptive pressures [53], or high levels of genetic drift in population bottlenecks [54]. These processes could increase the rate of fixation on non-synonymous substitutions, which may be reflected in an increase in ω (dN/dS) [52,55,56].

Two studies on large numbers of phylogenies found a recurrent correlation between root-to-tip distances and the number of speciation events [10,13]. This result was interpreted as evidence that clades with more speciation events have a faster rate of molecular evolution, which they attributed to punctuational change associated with the founder-effect model of speciation. However, while these phylogenetic tests reveal an association between rates of evolution and number of phylogenetic nodes, they are not able to localise those changes to the nodes rather than the edges of the phylogeny, so cannot distinguish between two alternative explanations, that speciation events increase the substitution rate or that higher substitution rate increases diversification. One possible way to separate these models is in their predicted effects on the patterns of substitutions. If population divisions associated with speciation events have significant effects on rates of substitution, either through change in selection or reduction in effective population size, it should result in a relative increase in the nonsynonymous rate, reflected in an increase in dN/dS (ω).

We did not detect any association between ω and net diversification (see also [8]). This may be because net diversification is not associated with consistent effects on population size [46], or diversification does affect effective population size, but the effect on ω is overwritten by other population fluctuations [46]. Alternatively, the effect on reduction in effective population size may be too small to be detected or may be affected by the method of estimation of ω [57]. In theory, *N*_*e*_ is an adequate representation of genetic drift in large populations and when the population size has been consistent for a long enough time [52]. It has even been shown that following transient increases in *N*_*e*_ there can be an increase in the rate of substitutions due to slightly advantageous mutations, which is the opposite of the predicted effect [58]. Therefore, although *N*_*e*_ is likely to have a significant effect on the rate of substitutions, predicting the form of the effect is far from a simple task [56]. Therefore, failure to detect an effect of population size changes of ω in this study does not imply that *N*_*e*_ is unaffected by diversification; however, it does suggest that changes in *N*_*e*_ during diversification are unlikely to explain the differences in substitution rates that we observe in these data.

### Molecular evolution and diversification in plants

Many studies have focussed on identifying the genetic loci underlying speciation. These can be genes that contribute to the genetic isolation of populations, genes that drive differential ecological adaptation, and “magic traits” that do both (e.g. [59,60]). Genome-wide scans are increasingly being used to identify outlier loci that show signatures of selection, including loci that differ between pairs that are associated with floral traits, climatic factors, and sterility [61]. This study takes a different, and complementary, approach to analysing the role that the genome-wide generation of genetic change plays in diversification.

It is possible that higher mutation rates may create a greater pool of standing variation from which adaptive substitutions can be derived. The assumption that chloroplast genes do not play a direct role in ecological adaptation has now been challenged: for example, values of ω above 1 have been estimated in rbcL [62] and MatK sequences [63] for some linages, which was interpreted as a signal of positive selection. However, we did not find evidence of higher ω in more diverse clades for the loci analysed in this study, and the relationship between the amount of standing variation and diversification in plants is not clear. For example, studies have found that diversification in orchids is not associated with greater genetic diversity at the population level [20,64].

Another scenario is that higher mutation rates contribute to the rate at which the genomes of different populations diverge and become gradually incompatible, making hybrids between the populations less fit. Bateson-Dozhansky-Muller (BDM) incompatibilities may arise from selection in different populations, but they might also be unconnected to ecological or behavioural divergence, in other words they may be “incidental on other acquired differences” [65]. For a mutation to go to substitution in one population, it must be broadly compatible with other common alleles in that population. But it will not have been “tested by natural selection” against alleles in isolated populations, and bringing those unharmonised alleles together may result in a maladapted individual [66]. The more unique substitutions each population has acquired, the greater the chance that a hybrid zygote will contain at least one pair of incompatible alleles. The steady increase in hybrid incompatibility with time in many species has been taken as evidence that many loci may contribute to BDM incompatibilities [47]. Under the BDM model, the rate of speciation may increase as the mutation rate increases [67]. Since the substitutions underlying BDM incompatibilities do not have to occur evenly in both lineages, a higher mutation rate in one lineage should drive divergence between them [47]. Debate continues over the rate at which hybrid incompatibility accumulates, particularly concerning the prediction that BDM incompatibilities should “snowball”, accelerating relative to the substitution rate [68,69].

Importantly, incompatibilities between populations can involve both organelle and nuclear genomes. Just as alleles within the nuclear genome must be able to work together to produce viable offspring, genomes of chloroplasts must be co-adapted to nuclear genome to allow normal development [70]. For example, alleles that cause cytoplasmic male sterility may be countered by suppression genes in the nuclear genome that restore male function, so a hybrid that inherited the organelle genome without the corresponding nuclear allele would be male sterile [33]. While cytonuclear conflict has been more frequently studied between mitochondrial and nuclear genomes, there is increasing evidence that incompatibilities between chloroplast and nuclear genomes contribute to hybrid incompatibility in many plant species [26].

Polyploidy is another important factor in the diversification of many plant lineages [71,72], but by focussing only on the chloroplast we minimized the impact of genome duplication on our analyses. Chloroplasts typically have uniparental inheritance, which simplifies the interpretation of the effects of genetic changes on divergence. However, the mode of inheritance of chloroplasts, whether inherited paternally or maternally, can vary between taxa, which can influence their levels of genetic diversity [73]. Chloroplast sequences should also limit the impact of “divergence hitchhiking”, where linked neutral loci go to fixation through being linked to a locus under selection [61]. Lastly, while chloroplasts use recombination for genome repair [74], hybridization of chloroplasts from different lineages does not appear to be common [26,75].

## Conclusions

We show a significantly faster rate of molecular evolution in chloroplast genes of species-rich lineages of the family Proteaceae. These results offer evidence for the influence of the rate of molecular evolution on diversification. This does not imply that the rate of molecular evolution explains the process of diversification, because this complex and heterogeneous process can be influenced by many mechanisms such as hybridization [76], polyploidy [77], allopatric events [14], and the duplication of genes [30]. However, the results do suggest that the substitution rate in chloroplasts may be one of these influences on the speed at which populations diverge, thus influencing the probability of populations becoming separate species [16].

## Methods

### Sister pairs

The phylogenetic analysis of the family Proteaceae by Sauquet et al. (2009) includes the 79 recognized genera and the species-richness for each genus compiled from the literature (references are also available in [78]). For the present study, monophyletic pairs of sister lineages that display differences in current species-richness were chosen from Sauquet et al’s phylogeny. The main criterion to select pairs was that the pair was monophyletic, so the two sister lineages had the same amount of time to accumulate species diversity and substitutions, and each sister pair was phylogenetically independent from all other such pairs.

We chose one genus to represent each sister lineage in order to avoid bias in branch length estimation due to the node density effect [79]. The chosen genus was the one with the greatest gene coverage. If the genera of a sister lineage had equal genetic coverage the genus was chosen at random. Using only one sequence per genus may reduce the power of the test, which may obscure a weak pattern; but using only one randomly selected species per sister lineage is unlikely to generate any systematic biases in rates, making this approach conservative for testing an association between rates of molecular evolution and net diversification (see [8,11]). In some cases a sister lineage is represented by combining sequences from several closely related genera. This practice increases our power to resolve the shared history of that lineage since its divergence from the common ancestor of the sister pair, and this is unlikely to create any systematic biases in rate estimations (Additional file 1: Table S1).

### Molecular dataset

Branch length estimation was critical for comparative analyses in this study, so the genetic dataset required unambiguous genetic alignments and the maximum gene coverage of the species analysed. With these criteria we included six genes of chloroplast origin (*atpB*, *atpB-rbcL*, *matK*, *rbcL*, *trnL* intron, and *trnL-trnF*) from the data by Sauquet et al. 2009 and available in the GenBank repository (Additional file 1: Table S1). These were then aligned using the MUSCLE algorithm, checked by eye, and manually corrected using the program SeaView v4 [80]. This resulted in a 6278bp alignment with 62 taxa, 4457bp of exons, and 1821bp of introns (Additional file 1: Table S2).

### Phylogenetic estimation

**Figure 1:**
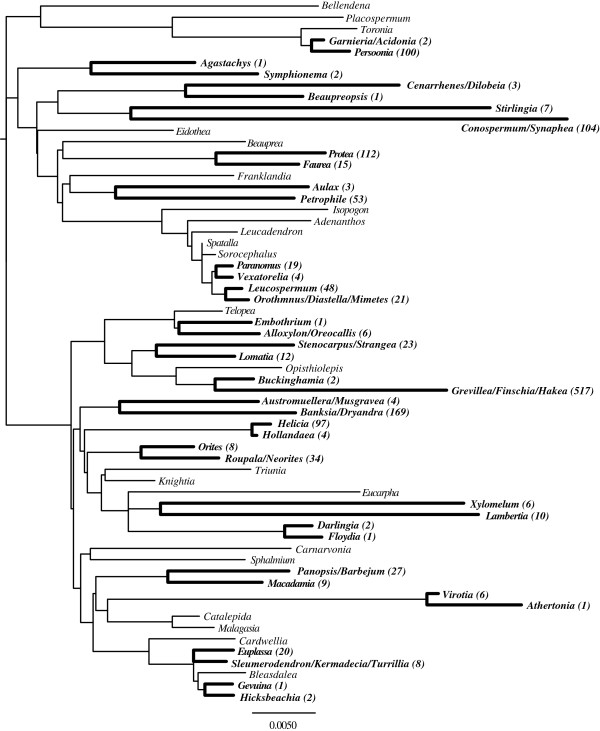
**Phylogeny of Proteaceae with the sister pairs analysed in bold.** Detailed legend: Molecular phylogeny of the family Proteaceae including the 20 sister pair groups used for the present analyses in bold. The branch lengths shown are proportional to the estimated number of substitutions as indicated by the whole dataset of six chloroplast genes. The species richness of the clades compared is shown in parentheses, with species numbers taken from Sauquet et al. (2009; references are also available in [78]). The scale bar indicates number of mutations.

Each gene alignment was tested for the most appropriate model of substitutions using likelihood estimation and comparison with the Bayesian Information Criterion as implemented in the package “ape” [81] in R (The R Project - http://www.r-project.org/). Applying a partition by genes with the models selected (Additional file 1: Table S2), a maximum likelihood analysis with 1000 replicates was run using Garli v2.0 [82]. The resulting tree was then used to extract the branch length values of the sister pairs (Figure 1). If any of our chosen sister pairs were not monophyletic in our phylogeny they were excluded from the analysis. Twenty sister pairs of the initial twenty-two chosen from Sauquet at al’s phylogeny were monophyletic in our estimates (Figure 1).

The inferred phylogenies (including those inferred for synonymous and non-synonymous substitutions; see next section) were examined for significant overall variation in branch lengths. To do this, we estimated the likelihoods of both a constant rates model and a free rates model (where there is one rate per branch) in the program HyPhy v2.1 [83], and compared them using a likelihood ratio test. If the free rates model provides a better fit for the data, this suggests significant variation in rates of molecular evolution across the phylogeny.

### dN and dS trees

To examine the potential link between synonymous substitutions (dS), non-synonymous substitutions (dN), and ω (dN/dS) and species-richness, we estimated dN and dS rates using an alignment of the coding genes (*atpB*, *matK*, and *rbcL*) in the program HyPhy v2.1 [83] with the MG94 model of codon evolution [84]. For the estimation of dN and dS trees, the MG94 model can be combined with any of the nucleotide substitution models nested in GTR + G + I. To choose the best combination we first gave HyPhy v2.0 a notation to estimate the codon frequencies (the frequency of each of the four bases in each of the three codon positions), which was a 3x4 matrix. Then the likelihood of each of the 203 possibilities was estimated and one was chosen according to the Akaike Information Criterion. The model chosen had four parameters, where θ_AG_ = θ_CT_ and θ_CG_ = θ_GT_, and its label in HyPhy v2.0 is MG94_3x4_012313. Finally, these parameters were optimized with maximum likelihood, constraining the topology to that estimated from the full six-gene dataset (Figure 1). The output included the dN and dS branch lengths (Additional file 2: Figure S1 and Additional file 3: Figure S2 respectively), which were used to extract the branch lengths of sister pairs. Given that HyPhy v2.0 estimates the values of dN and dS as the expected number of substitutions per nucleotide per site, the values for ω were calculated as the ratio between the two estimates (dN/dS; Additional file 4: Figure S3).

### Statistics

The total species-richness and the estimates of branch lengths (for all substitutions, dN and dS) and ω were collected for each of the two lineages in the sister pairs (Table 1). As the sister lineages had the same amount of time to accumulate species and substitutions, we assumed that the branch length is proportional to the rate of molecular evolution of chloroplasts (reviewed in [34]). Similarly, we assumed that species-richness of each sister clade reflects the net diversification (speciation minus extinction) of that lineage since the last common ancestor of the sister pair.

We performed a one-tailed Wilcoxon Signed-Ranks test in R, which resembles the standard sign test but accounts for the magnitudes of the differences between matched lineages [85]. This test sets a sign to each pair by subtracting branch lengths in the direction from species-rich to species poor; we did not include the sister pairs with equal species-richness as these cannot be accommodated in the Wilcoxon Signed-Ranks test. Then, the absolute difference between the two values was used to rank the pairs (lowest difference has rank 1 and the highest rank is the number of pairs). Tied values receive as a rank the mean of the ranks they span. The ranks are then given the sign of the pair and then added to produce a W statistic [85].

## Availability of supporting data

The dataset supporting the results of this article is publicly available in the GenBank repository, with the accession numbers listed in the Additional file 1: Table S1. Although the data was produced by several sources, it is summarized in Sauquet et al. (2009).

## Competing interests

Neither of the authors has received reimbursement, fees, funding, or salaries from any organisation that may gain or lose financially from the publication of this manuscript either now or in the future. This work was funded by the Australian Research Council. Neither of the authors do holds stocks or shares in an organisation that may gain or lose financially from the publication of this work either now or in the future. Neither of the authors hold or are currently applying for any patents related to this manuscript, nor have we received any reimbursements, fees, funding, or salaries related to patents for this publication. There are no financial or non-financial competing interests related to this manuscript.

## Authors’ contributions

David Duchene: ES & FG. Lindell Bromham: ES & FG. ES: Participated in the design of the study and performed statistical analyses. FG: conceived of the study, and participated in its design and coordination and helped to draft the manuscript. All authors read and approved the final manuscript.

## Supplementary Material

Additional file 1: Table S1 Species and GenBank accession numbers used in the present study. Species names are present beside the accession number if sequences for more than one species were used for the same lineage. **Table S2.** The chloroplast genes used in this study with the final alignment length and the substitution model selected. Click here for file

Additional file 2: Figure S1 Molecular phylogeny of the family Proteaceae that highlights the 20 sister pair groups used for the present analyses. The branch lengths and scale bars are proportional to the number of non-synonymous substitutions. Click here for file

Additional file 3: Figure S2 Molecular phylogeny of the family Proteaceae that highlights the 20 sister pair groups used for the present analyses. The branch lengths and scale bars are proportional to the number of synonymous substitutions. Click here for file

Additional file 4: Figure S3 Molecular phylogeny of the family Proteaceae that highlights the 20 sister pair groups used for the present analyses. The branch lengths and scale bars are proportional to the dN/dS branch lengths calculated from the estimates of trees for dN and dS (Additional file 2: Figure S1 and Additional file 3: Figure S2). Click here for file
