# Supplementary material for: Rates of molecular evolution and diversification in plants: chloroplast substitution rates correlate with species-richness in the Proteaceae
Source: BMC Evol Biol. 2013 Mar 13;13:65. doi: 10.1186/1471-2148-13-65 (PMC3600047; doi:10.1186/1471-2148-13-65)
Supplement: Additional file 1: Table S1 — Species and GenBank accession numbers used in the present study. Species names are present beside the accession number if sequences for more than one species were used for the same lineage. Table S2. The chloroplast genes used in this study with the final alignment length and the substitution model selected. [file 1471-2148-13-65-S1.docx]

Supporting Information – Research article

Title: Rates of molecular evolution and diversification in plants: chloroplast mutation rates correlate with species richness in the Proteaceae

Authors: David Duchene, Lindell Bromham

Table S1. Species and GenBank accession numbers used in the present study. Species names are present beside the accession number if sequences for more than one species were used for the same lineage.

| Comparison | Clade | Sequence | *atpB* | *atpB-rbcL* | *matK* | *rbcL* | *trnLi* | *trnL-trnF* |
| --- | --- | --- | --- | --- | --- | --- | --- | --- |
| 1 | Persoonia | *Persoonia spp.* |  |  | *P. linearis* EU169654 | *P. falcata* EU676075 |  |  |
|  | Garnieria + Acidonia | *Garnieria spathulaefolia* |  |  | EU169628 | EU676113 |  |  |
| 2 | Symphionema | *Symphionema montanum* | AF060394 | AF060733 | EU169667 | DQ875825 |  |  |
|  | Agastachys | *Agastachys odorata* | AF060393 | AF060717 | EU169607 | DQ875824 |  |  |
| 3 | Cenarrhenes + Dilobeia | *Cenarrhenes nitida* | AF060396 | AF060746 |  | DQ875827 |  |  |
|  | Beaupreopsis | *Beaupreopsis paniculata* |  |  |  | EU642641 | EU676044 | EU676048 |
| 4 | Conospermum + Synaphea | *Conospermum spp.* | *C. mitchelli* AF060398 | *C. mitchelli* AF060728 | *C. taxifolium* EU169617 | *C. mitchelli* DQ875829 |  |  |
|  | Stirlingia | *Stirlingia latifolia* | AF060397 | AF060738 | EU169666 | DQ875828 |  |  |
| 5 | Protea | *Protea cynaroides* | DQ875866 | AJ699186 | EU169658 | DQ875837 | AJ698243 | AJ698150 |
|  | Faurea | *Faurea spp.* |  | *F. macnaughtonii* AJ699262 | *F. forficuliflora* EU169625 | *F. saligna* EU676072 | *F. macnaughtonii* AJ698320 | *F. macnaughtonii* AJ698228 |
| 6 | Petrophile | *Petrophile spp.* | *P. circinata* AF060401 | *P. circinata* AF060735 | *P. canescens* EU169655 | *P. biloba* DQ875832 |  |  |
|  | Aulax | *Aulax spp.* |  | *A. umbellata* AF060732 | *A. umbellata* EU169610 | *A. cancellata* DQ875863 | *A. umbellata* EU676043 | *A. umbellata* EU676047 |
| 7 | Paranomus | *Paranomus spp.* |  |  | *P. bracteolaris* EU169653 | *P. reflexus* EU676074 |  |  |
|  | Vexatorella | *Vexatorella alpina* |  |  | EU169672 | EU676079 |  |  |
| 8 | Leucospermum | *Leucospermum spp.* |  |  | *L. pedunculatum* EU169642 | *L. bolusii* AM235083 |  |  |
|  | Orothamnus + Diastella + Mimetes | *Mimetes spp.* |  |  | *M. hirtus* EU169647 | *M. hottentoticus* EU676073 |  |  |
| 9 | Alloxylon + Oreocallis | *Alloxylon spp.* | *A.wickhamii* AF060428 | *A.wickhamii* AF060752 | *A. flammelum* EU169608 | *A. flammelum* DQ875856 |  |  |
|  | Embothrium | *Embothrium coccineum* | AF060429 | AF060754 | EU169622 | DQ875857 | AM397162 |  |
| 10 | Stenocarpus + Strangea | *Stenocarpus salignus* | AF060431 | AF060743 | EU169664 | DQ875859 | AF482149 | AF482194 |
|  | Lomatia | *Lomatia spp.* | *L. myricoides* AF060430 | *L. myricoides* AF060722 | *L. silaifolia* EU169644 | *L. silaifolia* U79171 | *L. silaifolia* AF482143 | *L. silaifolia* AF482188 |
| 11 | Grevillea + Finschia + Hakea | *Grevillea spp.* | *G. baileyana* AF060434 | *G. baileyana* AF060747 | *G. robusta* EU169631 | *G. robusta* AF197589 | *G. banksii* AM397163 | *G. banksii* AM397163 |
|  | Buckinghamia | *Buckinghamia spp.* | *B. celissima* AF060433 | *B. celissima* AF060742 | *B. ferruginiflora* EU169614 | *B. ferruginiflora* DQ875861 | *B. celissima* AF482145 | *B. celissima* AF482190 |
| 12 | Virotia | *Virotia leptophylla* |  |  |  | EU676122 |  |  |
|  | Athertonia | *Athertonia diversifolia* |  |  | EU169609 | EU676108 |  |  |
| 13 | Panopsis+ Brabejum | *Panopsis spp.* | *P. ferruginea* AF060421 | *P. ferruginea* AF060756 | *P. yolombo* EU169652 | *P. cinnamomea* DQ875850 |  |  |
|  | Macadamia | *Macadamia spp.* | *M. integrifolia* AY837827 | *M. jansenii* AF060750 | *M. integrifolia* AY823204 | *M. ternifolia* U79172 | *M. integriflora* AF482140 | *M. integrifolia* AF482185 |
| 14 | Hicksbeachia | *Hicksbeachia pinnatifolia* |  |  | EU169636 | EU676115 |  |  |
|  | Gevuina | *Gevuina avellana* |  |  |  | DQ875852 |  |  |
| 15 | Euplassa | *Euplassas occidentalis* |  |  |  | EU676051 | EU676054 | EU676059 |
|  | Sleumerodendron + Kermadecia + Turrillia | *Sleumerodendron austrocaledonicum + Kermadecia pronyensis* |  |  |  | EU676116 | EU676057 | EU676062 |
| 16 | Banksia + Dryandra | *Banksia spp.* | *B. ericifolia* AY837809 | *B. cuneata* AF060731 | *B. ericifolia* AY823186 | *B. ericifolia* DQ875843 | *B. ericifolia* AF482126 | *B. ericifolia* AF482171 |
|  | Austromuellera + Musgravea | *Austromuellera trinervia* | AY837825 | AF060720 | AY823202 | DQ875865 | AY823214 | AY823219 |
| 17 | Roupala + Neorites | *Roupala montana + Roupala monosperma + Neorites kevediana* | *N. kevediana* AF060411 | *N. kevediana* AF060716 | *R. montana* EU169661 | *R. monosperma* EU676052 | *R. montana* AF482144 | *R. montana* AF482189 |
|  | Orites | *Orites spp.* | *O. lancifolia* AF060412 | *O. lancifolia* AF060718 | *O. excelsa* EU169650 | *O. myrtoidea* DQ875842 | *O. lancifolia* AF482142 | *O. lancifolia* AF482187 |
| 18 | Darlingia | *Darlingia darlingiana* |  |  | EU169618 | EU676110 |  |  |
|  | Floydia | *Floydia praealta* | AF060416 | AF060713 | EU169626 | DQ875845 | AF482147 | AF482192 |
| 19 | Lambertia | *Lambertia spp.* | *L. formosa* AF060417 | *L. formosa* AF060737 | *L. formosa* EU169639 | *L. echinata* DQ875846 |  |  |
|  | Xylomelum | *Xylomelum spp.* | *X. scottianum* AF060418 | *X. scottianum* AF060741 | *X. angustifolium* EU169673 | *X. pyriforme* DQ875847 |  |  |
| 20 | Helicia | *Helicia spp.* | *H. australasica* AF060425 | *H. australasica* AF060724 |  | *H. sp*. DQ875853 |  |  |
|  | Hollandaea | *Hollandaea riparia* | AF060426 | AF060751 |  | DQ875854 |  |  |

Table S2. The chloroplast genes used in this study with the final alignment length and the substitution model selected.

| Gene | atpB | atpB-rbcL | matK | rbcL | trnLi | trnL-trnF |
| --- | --- | --- | --- | --- | --- | --- |
| Substitution model chosen | HKY+G | GTR+G | GTR+G | HKY+G+I | HKY+G | GTR+G |
| Alignment length | 1497 | 860 | 1791 | 1406 | 554 | 407 |
| Exon/Intron | Exon | Intron | Exon | Exon | Intron | Intron |

Figure S1. Molecular phylogenies of the family Proteaceae that highlight the 20 sister pair groups used for the present analyses. The branch lengths and scale bars are proportional to (a) the number of non-synonymous substitutions, (b) the number of synonymous substitutions, and (c) the dN/dS branch lengths calculated from the estimates of trees (a) and (b).
