# Supplementary figures and images for: Rates of molecular evolution and diversification in plants: chloroplast substitution rates correlate with species-richness in the Proteaceae
Source: BMC Evol Biol. 2013 Mar 13;13:65. doi: 10.1186/1471-2148-13-65 (PMC3600047; doi:10.1186/1471-2148-13-65)

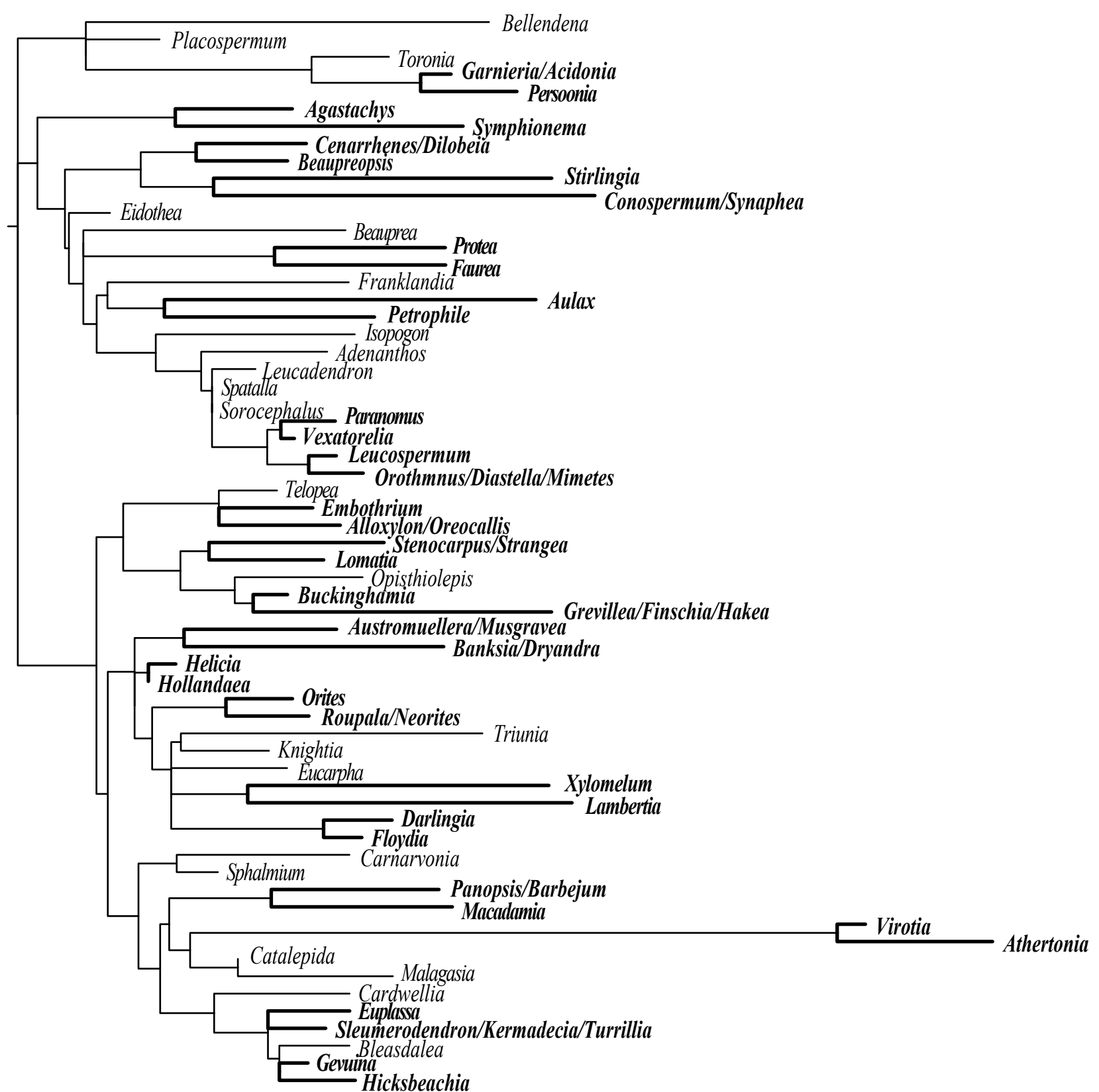

0.0030

Supplement: Additional file 2: Figure S1 — Molecular phylogeny of the family Proteaceae that highlights the 20 sister pair groups used for the present analyses. The branch lengths and scale bars are proportional to the number of non-synonymous substitutions. [file 1471-2148-13-65-S2.pdf]

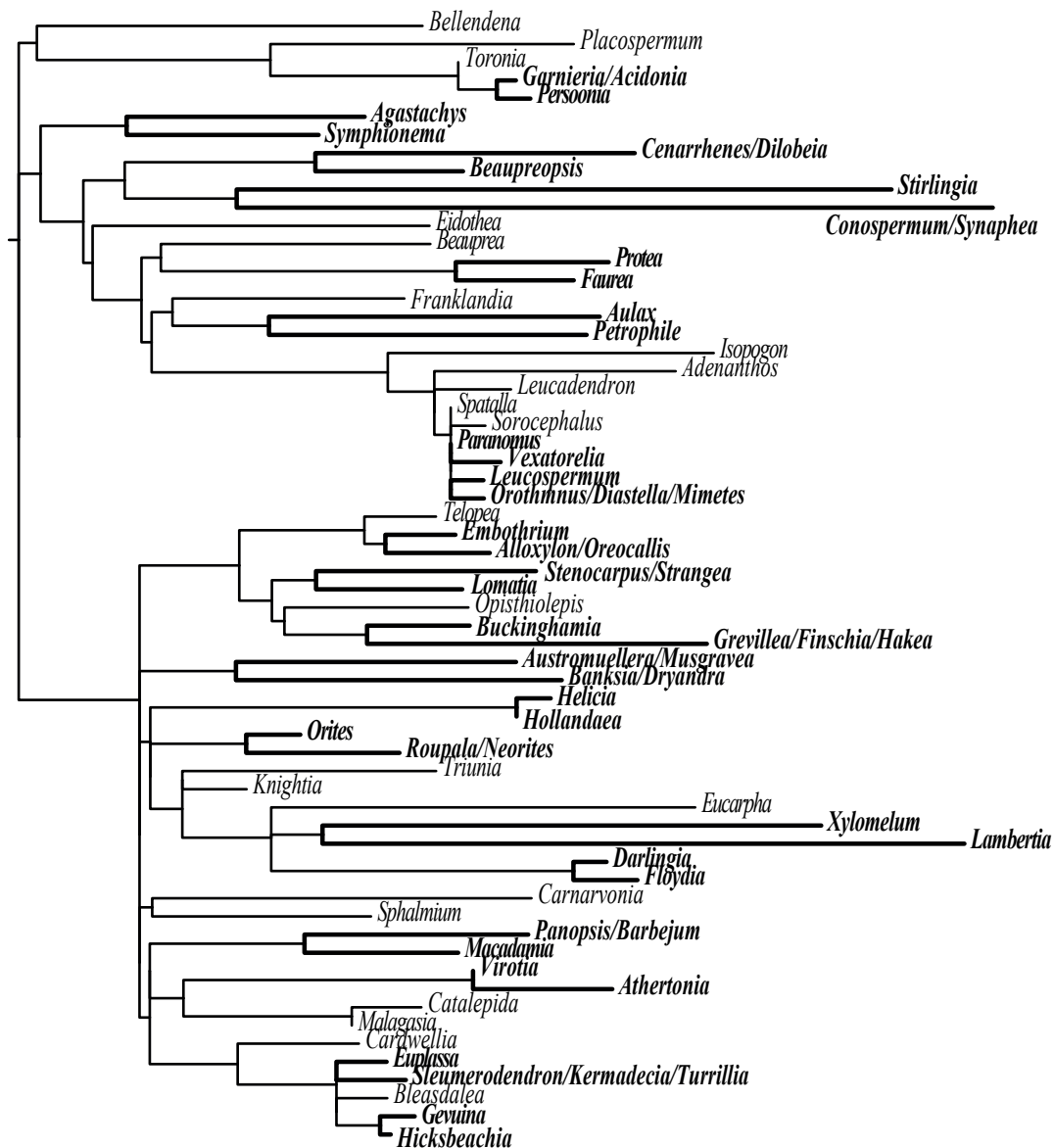

0.0090

Supplement: Additional file 3: Figure S2 — Molecular phylogeny of the family Proteaceae that highlights the 20 sister pair groups used for the present analyses. The branch lengths and scale bars are proportional to the number of synonymous substitutions. [file 1471-2148-13-65-S3.pdf]

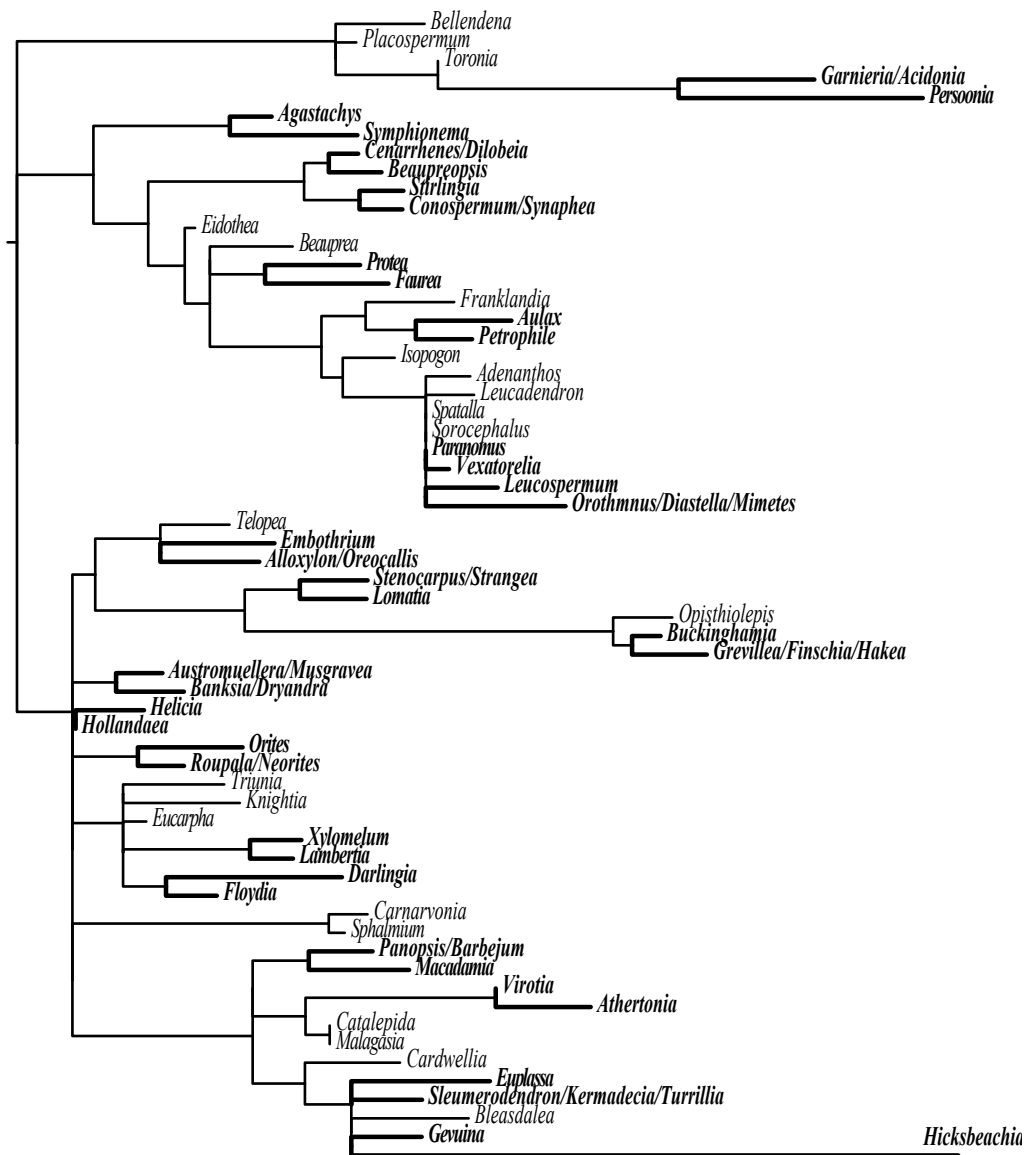

Supplement: Additional file 4: Figure S3 — Molecular phylogeny of the family Proteaceae that highlights the 20 sister pair groups used for the present analyses. The branch lengths and scale bars are proportional to the dN/dS branch lengths calculated from the estimates of trees for dN and dS (Additional file 2: Figure S1 and Additional file 3: Figure S2). [file 1471-2148-13-65-S4.pdf]
